# Structural characterization of four different naturally occurring porcine collagen membranes suitable for medical applications

**DOI:** 10.1371/journal.pone.0205027

**Published:** 2018-10-03

**Authors:** Thimo Maurer, Michael H. Stoffel, Yury Belyaev, Niklaus G. Stiefel, Beatriz Vidondo, Susanne Küker, Helga Mogel, Birgit Schäfer, Jasmin Balmer

**Affiliations:** 1 Division of Veterinary Anatomy, Vetsuisse Faculty, University of Bern, Bern, Switzerland; 2 Geistlich Pharma AG, Wolhusen, Switzerland; 3 Veterinary Public Health Institute, Vetsuisse Faculty, University of Bern, Bern, Switzerland; University of Insubria, ITALY

## Abstract

Collagen is the main structural element of connective tissues, and its favorable properties make it an ideal biomaterial for regenerative medicine. In dental medicine, collagen barrier membranes fabricated from naturally occurring tissues are used for guided bone regeneration. Since the morphological characteristics of collagen membranes play a crucial role in their mechanical properties and affect the cellular behavior at the defect site, in-depth knowledge of the structure is key. As a base for the development of novel collagen membranes, an extensive morphological analysis of four porcine membranes, including *centrum tendineum*, *pericardium*, *plica venae cavae* and *small intestinal submucosa*, was performed. Native membranes were analyzed in terms of their thickness. Second harmonic generation and two-photon excitation microscopy of the native membranes showed the 3D architecture of the collagen and elastic fibers, as well as a volumetric index of these two membrane components. The surface morphology, fiber arrangement, collagen fibril diameter and D-periodicity of decellularized membranes were investigated by scanning electron microscopy. All the membrane types showed significant differences in thickness. In general, undulating collagen fibers were arranged in stacked layers, which were parallel to the membrane surface. Multiphoton microscopy revealed a conspicuous superficial elastic fiber network, while the elastin content in deeper layers varied. The elastin/collagen volumetric index was very similar in the investigated membranes and indicated that the collagen content was clearly higher than the elastin content. The surface of both the *pericardium* and *plica venae cavae* and the cranial surface of the *centrum tendineum* revealed a smooth, tightly arranged and crumpled morphology. On the caudal face of the *centrum tendineum*, a compact collagen arrangement was interrupted by clusters of circular discontinuities. In contrast, both surfaces of the *small intestinal submucosa* were fibrous, fuzzy and irregular. All the membranes consisted of largely uniform fibrils displaying the characteristic D-banding. This study reveals similarities and relevant differences among the investigated porcine membranes, suggesting that each membrane represents a unique biomaterial suitable for specific applications.

## Introduction

As a structural protein with excellent biocompatibility, low antigenicity, pronounced cell affinity and biodegradability, collagen is a widespread biomaterial in regenerative medicine with the potential to regenerate tissues and restore their physiological function [1–4]. In dental medicine, decellularized biodegradable collagen membranes are widely used to create different compartments for defect healing. For guided bone regeneration (GBR), collagen membranes serve as an occlusive barrier to prevent the ingrowth of gingival soft tissue into a periodontal or bone defect, thus allowing tissue regeneration via the unhampered proliferation and differentiation of site-specific progenitor cells. These collagen membranes are derived from natural tissues such as porcine skin, human dermis or bovine Achilles tendon [4–8]. All connective tissues are composed of fibroblasts as a cellular component and of a highly organized extracellular matrix (ECM), which in turn comprises fibrous proteins, as well as an amorphous ground substance made of glycoproteins, growth factors and water [9]. The ECM, which is secreted by resident cells, serves as a physical support for cells, endows the tissue with its structural, mechanical and biochemical properties and controls cell behavior by cell-matrix interactions [10,11]. Within the ECM, collagens are the most abundant proteins [10,12]. To date, 28 different collagen types have been described and can be grouped into distinct classes based on their suprastructural organization [13]. The fibril-forming collagens (types I, II, III, V, XI, XXIV and XXVII) are the most widespread family of collagen types. These collagens constitute the main structural element of the ECM and provide the tissue with its tensile strength [13,14]. Collagen fibrils consist of covalently cross-linked collagen molecules measuring 300 nm in length. Partial overlap of collagen molecules results in a gap-overlap array, which produces the typical axial banding pattern named D-periodicity [10,13,15–17]. In transmission electron microscopy, the average D-periodicity is in the range of 64–70 nm [17]. Microfibrils or subfibrils are filamentous subunits of the collagen fibril [18–21]. Depending on tissue and age, the cylindrical collagen fibrils exhibit a diameter from 10 to more than 500 nm [22,23]. The length of collagen fibrils in mature tissue is difficult to estimate as fibril ends are rarely seen in micrographs. Presumably, collagen fibrils reach a length of several mm, or they even span the entire length in force-transmitting tissues such as tendons and ligaments [24]. Usually, parallel fibrils are interconnected by proteoglycans to form collagen fibers. Typically, fibers are cord- or tape-shaped, run in a wavy course and may reach a diameter up to 100 μm. However, the size and shape of collagen fibers depend on the tissue and organ [17].

Elastic fibers are another connective tissue element in extensible organs such as the arteries, lung, or skin. Elastic fibers endow the tissue with elasticity and resilience, thus allowing repeated stretch and the subsequent passive recoil [25,26].

Various membrane properties such as the diameter, spacing and orientation of fibers or fibrils, as well as membrane stiffness, have been reported to modulate cellular behaviors such as differentiation, migration and proliferation [27–31]. Since differences in collagen tissue structure lead to different biological responses and hence account for their specific potential in regenerative medicine, additional tissue sources of collagen membranes are of great interest. The goal of the present study, therefore, was to characterize four naturally occurring porcine membranes, i.e., c*entrum tendineum* (CT), *pericardium* (PE), *plica venae cavae* (PL) and *small intestinal submucosa* (SIS), with respect to their structural properties. Both sides of CT, PE and PL are covered by a mesothelium, whereas SIS is devoid of mucosal and muscular layers due to the manufacturing process [32]. Bovine pericardium is commonly used in regenerative medicine to replace heart valve leaflets and as a patch material in cardiac surgery [33,34], whereas SIS is used to reconstruct various soft tissues such as skin, vessels, the body wall or the urinary bladder [35,36]. CT has recently been considered a scaffold material [37,38]. The four selected collagen membranes were evaluated with respect to thickness, as this parameter is an important factor affecting the diffusion between blood capillaries and adjacent tissues. Qualitative assessment of the collagen ultrastructure and quantitative analysis of the collagen fibril diameter and D-periodicity was performed on NaOH-pretreated membranes using scanning electron microscopy (SEM) [39,40]. The collagen and elastic fiber organization in superficial and deeper tissue layers were visualized by second harmonic generation (SHG) and two-photon excited fluorescence (TPEF) microscopy, respectively. In addition to a qualitative structural analysis of SHG and TPEF micrographs, an elastin/collagen volumetric index was calculated. By providing detailed information on the thickness and structure of four different porcine membranes, this study is expected to pioneer the development of new collagen barrier membranes that may be tailored to specific clinical requirements. Overall, differences in membrane thickness and in their ultrastructure were detected, thus indicating that the four membranes might serve as raw materials for specific clinical applications.

## Materials and methods

### Membrane collection

Samples of CT, PE and PL were collected at a local abattoir (Metzgerei Nussbaum, 3114 Wichtrach, Switzerland) from approximately 6-month-old feeding pigs (≈140 kg) immediately after slaughter. Membranes were stored in phosphate buffered saline (PBS, pH 7.4) at ambient temperature during the transport to the laboratory. There, the membranes were rinsed in PBS to remove any blood before further processing or dry storage at -80°C.

SIS was purchased from Nikki SA (Boyaux Naturels, Switzerland), delivered in a pickling salt solution and stored at 4°C for several months. Prior to tissue processing, randomly selected pieces of SIS were rinsed in tap water for several hours. Considering that the assessed pieces were obtained from the intestines of different pigs or only from different locations of the same or several intestines, the term *piece* is used rather than *sample*.

### Thickness measurements

Thickness measurements were performed using a digital thickness dial gauge (Käfer Messuhrenfabrik GmbH & Co., Germany). Membranes were unfurled on a customized plastic platform, and the whole mount thickness was measured. The contact pressure was 1.2 kPa, and thickness values were read out once the viscoelastic changes had stabilized. Three distinct spots were selected per membrane, and triplicates were measured for every single spot to calculate the average membrane thickness. Spots that visually differed from the general appearance of the membrane because of local fat storage or large blood vessels were excluded. Eleven PBS-washed native samples of CT, PE and PL and fifteen pieces of SIS were measured. For CT, PE and PL, the samples were harvested from 33 different pigs so that only one of the three membranes was collected per pig.

### Scanning electron microscopy

Rinsed membranes were trimmed to a size of ca. 1 x 1 cm prior to fixation at 4°C overnight using 2.5% glutaraldehyde (Merck, Darmstadt, Germany) in 0.1 M sodium cacodylate buffer (pH 7.4; Merck) for at least 12 hours. Next, cells were removed by NaOH digestion according to [40]. Briefly, specimens were immersed in a 10% aqueous solution of NaOH for 3 to 6 days at room temperature and were then rinsed in distilled water for up to 24 hours. Thereafter, the specimens were dehydrated through an ascending ethanol series and critical point dried using an EM CPD 300 (Leica Microsystems, Heerbrugg, Switzerland). Dried specimens were halved with a razor blade and mounted onto aluminum stubs by means of double adhesive conductive tabs (Portmann Instruments, Switzerland) with the opposite sides facing up. The specimens were then sputtered with 15 nm of platinum in a Bal-Tec SCD 004 sputtering device (Bal-Tec AG, Balzers, Liechtenstein) and stored in a dessicator. SEM images were obtained with a DSM 982 Gemini digital field emission scanning electron microscope (Zeiss, Germany) at an accelerating voltage of 5 kV and a working distance of 6 mm.

SEM was performed on six samples of CT, PE and PL and six pieces of SIS. The samples of CT, PE and PL were harvested from six pigs. Therefore, from every pig, a sample of each of these three membrane types was collected. Images were acquired from two distinct spots per membrane face (recto/verso). From every spot, an image series was acquired at 200x, 1,000x, 5,000x, 10,000x and 50,000x magnifications. In addition to the qualitative and descriptive analysis of the collagen structure, the fibril diameters and D-periodicity were determined at 50,000x magnification for every membrane by means of the measuring tool integrated in the DSM 982. Fibril diameter was determined on a representative selection of fibers, avoiding the inclusion of obvious thick or thin fibers. D-periodicity was calculated by measuring the distance over several D-periods and subsequently dividing the value by their count. At minimum, 30 measurements for either of the two criteria were performed per membrane side.

### Second harmonic generation/two-photon excited fluorescence (SHG/TPEF)

Native membranes were stored at -80°C before imaging using second harmonic generation/two-photon excited fluorescence (SHG/TPEF) microscopy. After thawing at room temperature, the membranes were rinsed in PBS before excision of square specimens of 10 mm x 10 mm size using scissors. The specimens were then mounted on an object slide with a drop of PBS such that the opposite membrane sides were facing up, and the specimens were covered with a 0.17 mm thick glass cover slip (Glaswarenfabrik Karl Hecht GmbH & Co, Sondheim vor der Rhön, Germany). One-well Secure-Seal spacers (Thermo Fisher Scientific, Waltham, USA) were used to avoid squeezing the specimen.

SHG/TPEF images of the porcine membranes were acquired using a Leica TCS SP8 MP inverted multiphoton laser scanning microscope (Leica Microsystems, Germany) equipped with a Mai Tai XF (Spectra-Physics, USA) femtosecond Ti-sapphire pulsed laser, which was tunable from 720 to 950 nm. The excitation wavelength was set to 880 nm to excite both the broadband autofluorescence of elastin (500–650 nm) and the SHG signal of collagen (440 nm). A 25x/0.95 NA water immersion objective lens (HCX IRAPO L, Leica Microsystems, Germany) and a 0.55 NA condenser lens (Leica 0.55 S28 Number 505234) were used. A DAPI emission filter (435–485 nm) and a photomultiplier were used to record the forward SHG signal. The SHG signal in the epi-direction and the TPEF signal were both recorded by internal hybrid detectors (Leica Microsystems), with the detection bandwidth range set to 430–460 nm (SHG) and 500–650 nm (TPEF). The pinhole aperture was always fully open. Z-stacks of 1504 x 1504 pixel images (pixel size 0.2 x 0.2 μm) were acquired with a z-step size of 0.8 μm. Scanning of the samples was performed in bidirectional mode with a scanning speed of 600 Hz in combination with line averaging of 3 scans. Because of the different laser intensities required to generate the SHG and TPEF signals, recording of SHG and TPEF was performed in sequential mode by switching between frames. The laser power and detector gain were adjusted for each new image series based on visual assessment. Z-compensation was applied for all the image stacks.

Only CT, PE and PL were imaged using these methods as the pickling salt treatment prevented analysis of SIS by SHG/TPEF. Six samples per membrane type were investigated, and these samples were harvested from a total of six pigs. Z-stacks were acquired from two spots per membrane side. SHG/TPEF z-stacks were deconvolved using Huygens Remote Manager (Scientific Volume Imaging, Netherlands) before image processing with IMARIS software (Bitplane AG, Switzerland). Sectional images and 3D composite images of the deconvolved z-stacks were assessed in a qualitative manner, with descriptions of the collagen and elastin arrangements. Furthermore, an elastin/collagen volumetric index was calculated from two z-stacks for every sample of any given membrane type. For this purpose, forward and backward SHG channels were combined in Fiji software [41] prior to performing a separate surface rendering of the SHG and the TPEF channels using IMARIS. The volumetric index from the two surface-rendered channels was computed based on the volumes (v) of the collagen and elastin, as reported previously [42]: [V_Elastin_-V_Collagen_/V_Elastin_+V_Collagen_]. This calculation yielded indexes from +1 (100% elastin) to -1 (100% collagen).

### Statistical analysis

Statistical analysis was performed using NCSS 11 (NCSS, LLC, USA) for membrane thickness, D-period, volumetric index (elastin/collagen) and fibril diameter. Descriptive statistics revealed deviations from the normal distribution (Shapiro-Wilk test p<0.00) for the membrane thickness, fibril diameter and D-period but not for the volumetric index. Therefore, differences in the volumetric index among the membrane groups were assessed using one-way ANOVA, while differences in fibril diameter, D-period and membrane thickness were compared using the Kruskal-Wallis non-parametric one-way ANOVA and Dunn’s (all-pairwise) post hoc multiple comparison tests.

To assess differences between different samples (or between different pieces for SIS) of a given membrane type, the one-way ANOVA and Bonferroni multiple comparison tests were used for all outcome variables. All raw data as well as statistical descriptive outputs are accessible under https://doi.org/10.6084/m9.figshare.7058600.

The significance level was set to 0.05.

## Results

### Thickness measurements

**Fig 1:**
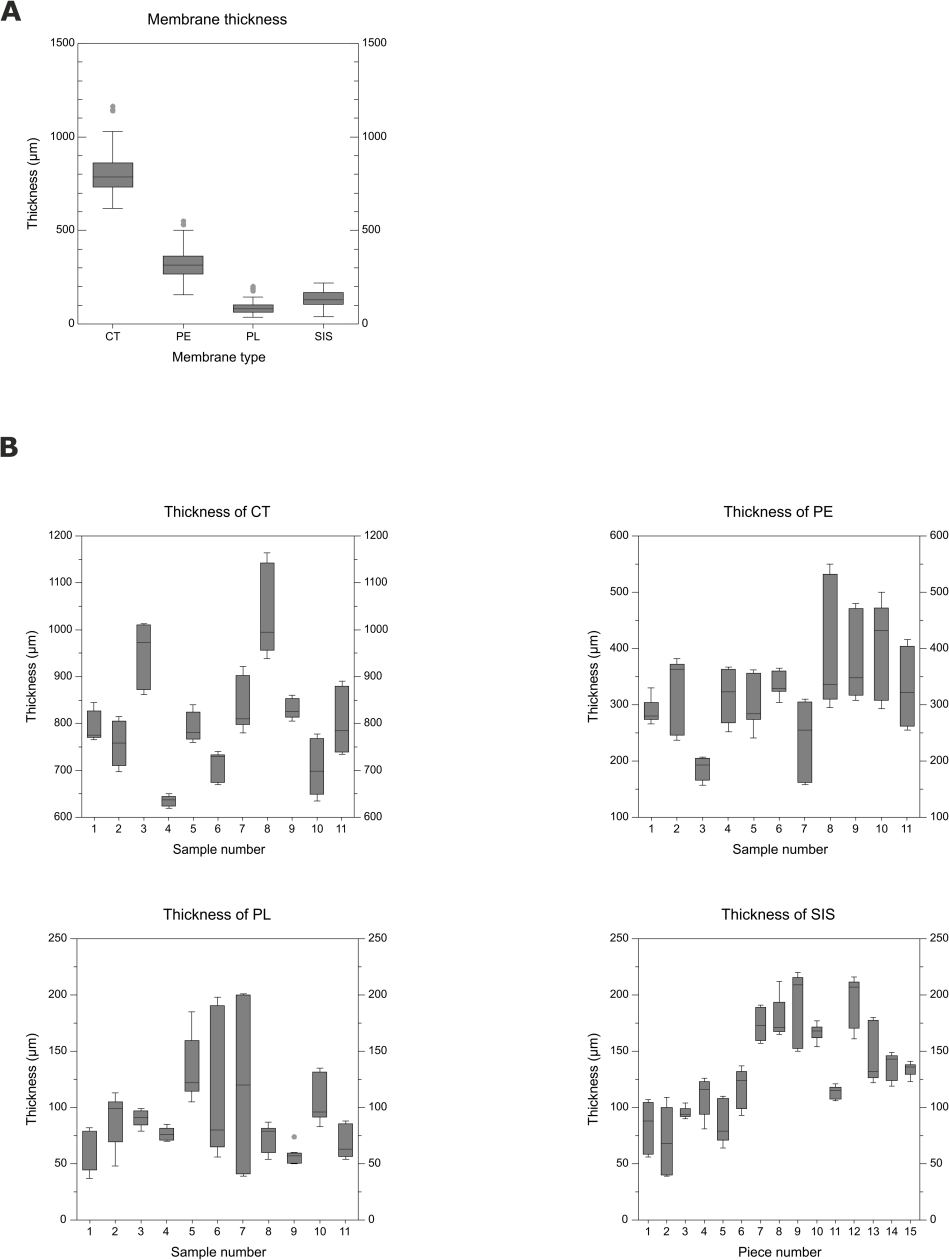
Box plots of membrane thickness measurements of native CT, PE, PL and SIS. (A) Membrane thickness was assessed on native membranes (CT, PE, and PL: N = 11; SIS: N = 15). For every sample of CT, PE and PL and for every piece of SIS, three triplicate measurements at three distinct spots were performed. The plica was the thinnest membrane, whereas CT was the thickest. Statistical comparison of the membrane types (Kruskal-Wallis one-way ANOVA and Dunn’s post hoc test) showed that all membrane types were significantly different from each other in terms of median thickness. (B) Box plots show the thickness measurements of all individual samples and pieces for every given membrane type. Statistical analysis showed significant differences within every membrane type. This result illustrates the considerable heterogeneity of the material.

The four porcine membranes (PL, PE, CT, and SIS) were assessed regarding their thickness in the native state. The obtained results are shown in Fig 1, and the detailed results are listed in Table 1.

**Table 1:** Thickness measurements of porcine membranes.

membrane	# of measurements	mean (μm)	median (μm)	SD (μm)	Q_1_ (μm)	Q_3_ (μm)
CT	99	804	785	118	733	860
PE	99	319	315	85	267	363
PL	99	90	82	38	63	103
SIS	135	133	130	44	104	168

For every membrane type, thickness was assessed at ≥ 33 locations (triplicates each), i.e., at 3 spots on each of the 11 (CT, PE, PL) samples or 15 (SIS) pieces. The Kruskal-Wallis one-way ANOVA test showed significant differences between all the membrane types.

Overall, the median thickness (Fig 1A) was 785 μm for CT, 315 μm for PE, 82 μm for PL and 130 μm for SIS. The differences between all studied membrane types were statistically significant for the membrane thickness. The differences between individual samples were also assessed (Fig 1B) and revealed that for each given membrane type, the median thicknesses of some of the samples differed significantly from each other. The median thicknesses ranged from 637–995 μm in CT, from 193–432 μm in PE and from 57–122 μm in PL (Fig 1A). The median thickness of different SIS pieces was in the range of 68–209 μm (Fig 1A), with significant differences occurring between the medians of some but not all collected pieces (Fig 1B), indicating a certain level of heterogeneity.

### SEM

#### Collagen structure

**Fig 2:**
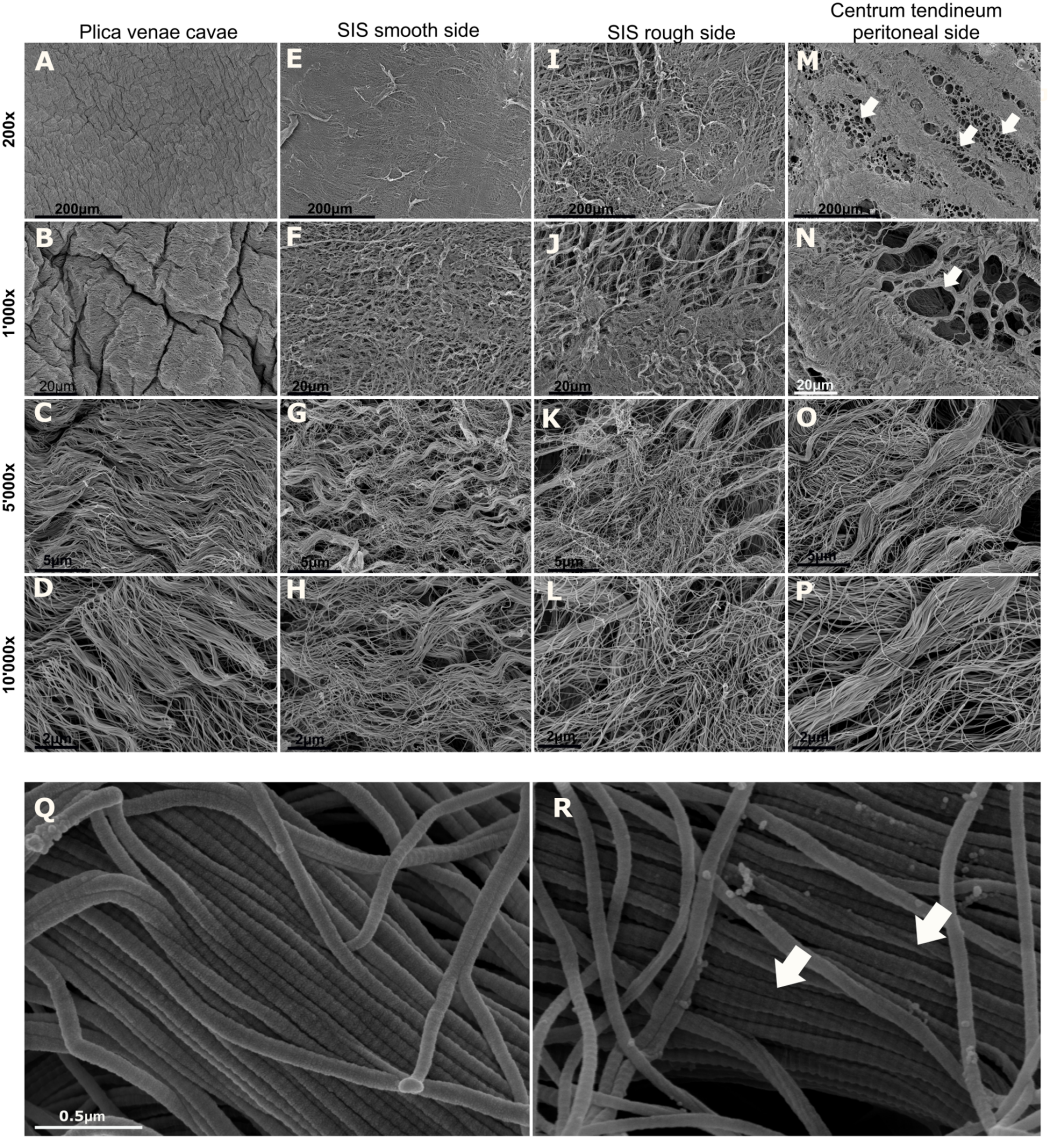
SEM micrographs of porcine collagen membranes. (A)-(D) show different magnifications (200x to 10,000x) of one sample obtained from the PL. The observed smooth surface morphology of PL is also detectable in PE and on the cranial face of CT. (E)-(H) show micrographs obtained from the smooth side of SIS. (I)-(L) are images obtained from the rough side of SIS, and (M)-(P) depict the magnified micrographs from the peritoneal (i.e., caudal) side of CT. The caudal side clearly shows circular discontinuities (arrows), whereas the cranial side does not (https://doi.org/10.6084/m9.figshare.7082999). (Q) High magnification of fibril bundles reveals the typical periodic banding pattern of collagen fibrils. (R) Collagen fibrils showing right-handed helical grooves (arrows) are occasionally found in all the membrane types.

SEM was performed to assess the superficial collagen structure of the porcine membranes after decellularization with NaOH (Fig 2). Both sides of the PE and PL membranes and the cranial face of the CT membrane displayed a smooth, compact and irregularly crumpled surface morphology, as shown in Fig 2A and 2B. Collagen fibrils formed fine, loosely arranged, undulating collagen bundles. The packing of fibrils into fibers was not very tight, resulting in single fibrils or small bundles interconnecting larger bundles. Isolated fibrils running crosswise formed a loose meshwork overlying the bundled fibrils (Fig 2C and 2D). Overall, bundles of different sizes were aligned in parallel and were separated from each other by irregular spaces. On the other hand, both sides of the SIS membrane displayed a fibrous and irregular appearance, with one face being smoother than the other (Fig 2E and 2I). Both membrane faces were lacking the crumpled appearance of the other membrane types. The fibers on the smoother side (Fig 2E–2H) of the SIS were thinner than those on the rougher side (Fig 2I–2L). As seen from the rough side, the different collagen layers consisted of reticulated fibers. A conspicuous loose meshwork of isolated fibrils was observed in the SIS membrane as well (Fig 2G). While the cranial side of the CT membrane resembled that of the PL and PE membranes (https://doi.org/10.6084/m9.figshare.7082999), the caudal surface of the CT membrane showed a particular pattern (Fig 2M). Here, the continuous surface pattern described above was interrupted by clusters of circular discontinuities that were often arranged in parallel stripes (Fig 2M and 2N, arrows).

In all the membranes studied, the collagen fibrils displayed the characteristic periodic D-banding pattern (Fig 2Q). Moreover, some fibrils revealed right-handed helical grooves (Fig 2R, arrows), which are indicative of supertwisted microfibrils. When bundled up, the collagen fibrils sometimes were intertwined with each other. Overall, the fibril shape was very homogeneous and uniform for a given membrane type except for rare outliers.

#### Fibril diameter

The median diameter of the collagen fibrils was 70 nm in CT, 56 nm in PE, 58 nm in PL and 47 nm in SIS. No statistically significant difference in fibril diameter was found between PE and PL (Dunn’s test), while all the other membrane type-based comparisons revealed statistically significant differences. When considering the corresponding membranes from different individuals, the median fibril diameters ranged from 60–81 nm in CT, 53–64.5 nm in PE and 50–64 nm in PL. The median diameter of collagen fibrils in SIS was in the range of 45–48.5 nm. Within all membrane types, some of the samples (or pieces for SIS) were significantly different from each other in terms of their median thickness (Kruskal-Wallis and Dunn’s test).

#### D-periodicity

The median length of the D-period in collagen fibrils was 53 nm in CT and PE, 52 nm in PL and 55 nm in SIS. With the Kruskal-Wallis and Dunn’s post hoc tests, there was no statistically significant difference detectable between CT and PE, while all the other membrane type-based comparisons revealed statistically significant differences. When comparing the D-period of different animals for each membrane type, the D-period median values ranged from 52–57 nm in CT, 52–55 nm in PE and 51–55 nm in PL. For SIS, the median D-period was in the range of 55–55.5 nm. The Kruskal-Wallis and Dunn’s post hoc tests revealed significant differences between some samples of each membrane type (CT, PE, and PL). Different pieces of SIS did not reveal significant differences in D-period.

### SHG/TPEF

#### Descriptive image analysis

**Fig 3:**
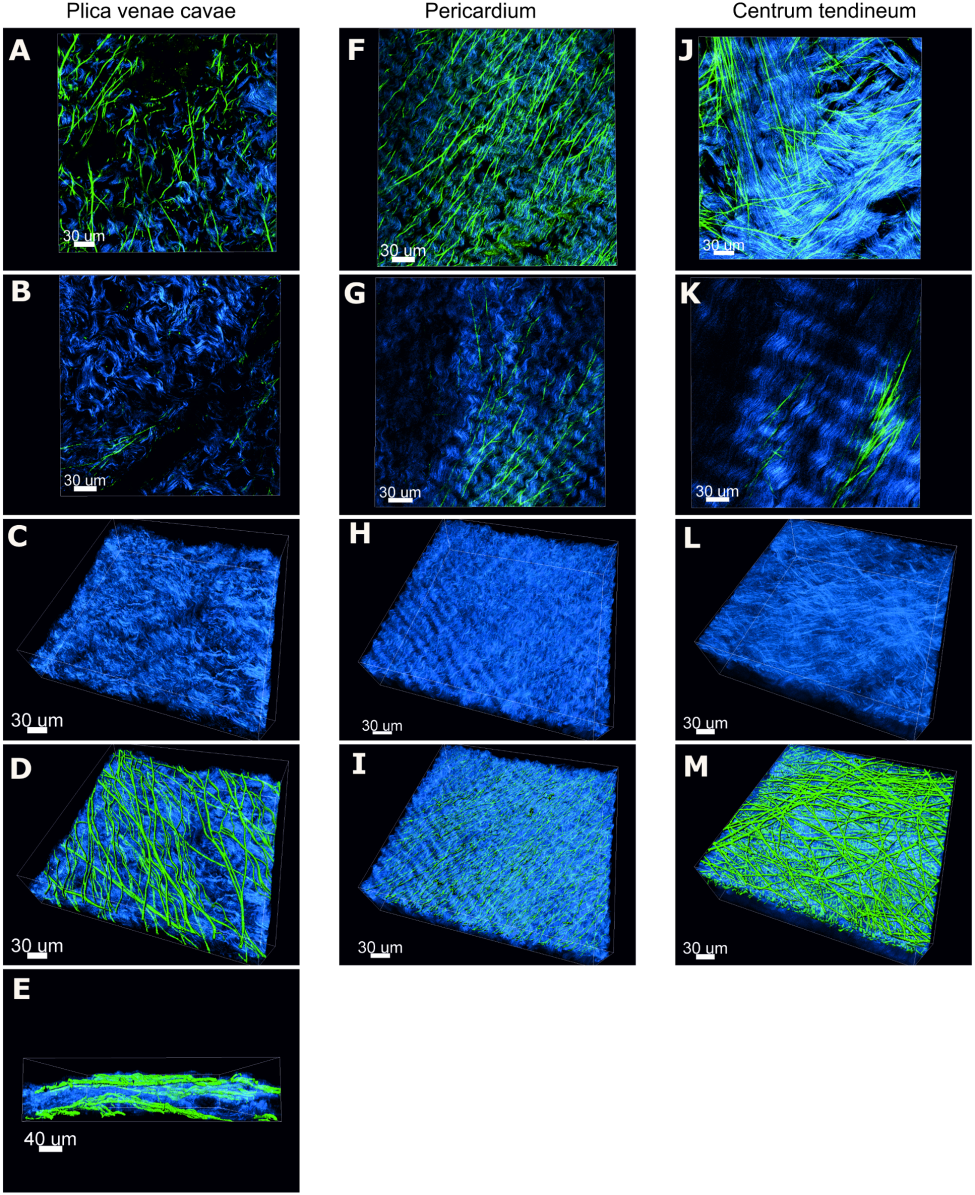
SHG and TPEF of native porcine membranes. Micrographs of single optical section planes and 3D composites of z-stacks generated by SHG/TPEF reveal the collagen and elastic fiber arrangement in PL, PE and CT. Collagen is shown in blue, while the elastic fibers appear in green (surface rendered). (A, F, J) are single optical section planes of a superficial tissue layer of the corresponding membrane type. The preferential orientation of the collagen fibers exhibits some variation. Elastic fibers are abundant in the superficial tissue layers. (B, G, K) are single optical section planes in deeper regions of the corresponding membrane type. The collagen alignment is variable again. Elastic fibers in deeper layers are frequently observed in PE and CT but are rare in PL. (C, H, L) are 3D composites of the collagen structure alone, showing undulating collagen fibers arranged in planes parallel to the membrane surface. (D, I, M) are the same 3D composites also showing the elastic fiber network. (E) is a cross-sectional view of the 3D composite of PL, revealing the sandwich-like structure arising from the superficial elastin network and the core of the membrane that is almost devoid of elastin.

The collection of both the backward and forward SHG signals allowed an increased imaging depth, which was particularly valuable in CT. The forward scattered SHG revealed finer and more defined collagen structures than did the backscattered signal. The TPEF signal, which was used to detect elastin, was generally less intense than the SHG signal. Membranes were analyzed in depth for collagen (depicted in blue, Fig 3) and elastin (color-coded in green, Fig 3) structure.

#### Plica venae cavae

Fig 3A–3E show different sections and views of the images obtained from PL. Overall, undulating collagen fibers formed a loose meshwork in parallel with the membrane surface, which occasionally showed a preferential fiber alignment. A favored fiber orientation was limited to discrete areas within individual collagen layers. A distinct network of frequently branching straight cylindrical elastic fibers was present in the two most superficial layers (recto-verso) of the membrane (Fig 3A). In the intermediate layers, elastic fibers were only sporadically seen (Fig 3B).

Fig 3C–3E show 3D views of the PL membrane with its elastin network and the undulating collagen backbone. These micrographs substantiate the notion that the PL membrane consists of a sandwich-like structure with inner layers being virtually devoid of elastin and superficial layers comprising substantial amounts of elastin.

#### Pericardium

Unfortunately, full-thickness imaging of the PE membrane was not feasible. Therefore, the samples were scanned from both sides to obtain as much information as possible. Collagen showed a particularly pronounced ripple in this membrane type. The collagen fibers were arranged in layers that paralleled the surface (Fig 3F–3G). The degree of preferential fiber orientation was variable. Elastic fibers took a straight course and underwent extensive branching. These fibers were most abundant in the superficial tissue layers, but their occurrence remained noticeable in deeper layers as well. Most elastic fibers ran parallel to the collagen fibers, although a few elastic fibers running crosswise were regularly seen, especially in the superficial layers.

#### Centrum tendineum

In the CT membrane, the ripple of the collagen was inconstant, and some collagen fibers displayed a mostly straight course (Fig 3J). In this membrane, the collagen fibers were also arranged in layers, which were parallel to the surface. Within these layers, the degree of collagen fiber orientation was variable. While some layers of collagen fibers typically showed less uniformity regarding collagen fiber orientation and thus resembled an interwoven fiber network (Fig 3J), others exhibited a higher degree of preferential fiber orientation (Fig 3K). The shape of the elastic fibers in both superficial (Fig 3J) and deeper tissue layers (Fig 3K) was cylindrical, and their course was straight with occasional branching. 3D images (Fig 3L and 3M) revealed a dense superficial elastin structure with several layers of elastic fibers with varying orientation. The elastic fibers were rare but still present in the deeper tissue layers.

#### Elastin/Collagen volumetric index

The volumetric index of elastin and collagen can vary between +1 and -1, which correspond to pure elastin and collagen, respectively (a value of -0.8 would indicate a collagen content of 90%). The mean elastin/collagen volumetric index was -0.78 ± 0.15 for CT, -0.75 ± 0.13 for PE and -0.67 ± 0.11 for PL. The volumetric indexes in our membranes account for a collagen content clearly exceeding the elastin content. No statistically significant differences in elastin/collagen volumetric indexes between different membrane types were observed using one-way ANOVA and Bonferroni multiple comparison tests.

## Discussion

The goal of the present study was to structurally characterize four porcine membranes that have the potential to serve as a raw material for the production of novel collagen barrier membranes. Corresponding tissues were selected on the basis of biological requirements such as appropriate thickness and on the basis of their suitability for further downstream processing, i.e., ease of harvest, abundance and homogeneity of the raw material.

As the slaughtering age (6 months) and weight (≈140 kg) of feeding pigs are fairly standardized, individual body weight and age were not taken into account as variables in the present study. Thus, minor variations in these parameters may be expected but are considered irrelevant to the aims of the study. For the commercially available SIS, no information about the number, age or weight of the pigs from which SIS was obtained was procurable. Furthermore, SIS was stored in saline prior to analysis, precluding its analysis by SHG microscopy. Consequently, the provenance and manufacturing must be kept in mind when interpreting the findings about SIS and when comparing SIS with the other three tissues.

Membrane thickness affects the mechanical properties of a membrane, defines the diffusion distance between tissue compartments and, therefore, provides a rationale for selecting membranes in view of specific applications in regenerative medicine. The average thicknesses of the *centrum tendineum* (CT), *pericardium* (PE) and *small intestinal submucosa* (SIS) as determined in our study were comparable to previous findings [32,37,43,44], whereas no data regarding the thickness of the *plica venae cavae* (PL) was available. CT, which had a thickness greater than 600 μm, may be predicted to be useful for laparoscopic hernia repair [37], whereas the delicacy of PL supports its use in the reconstruction of thin-walled hollow organs.

Beyond the average thickness, the range of variation within a given membrane type may also affect its suitability for the manufacture of products with defined specifications. Variations in thickness measurements for the different membrane types studied mainly arose from interindividual differences. Furthermore, considerable variation within individual samples was typical for CT and PE. While thickness changes were gradual in CT, inhomogeneity in PE resulted from discrete areas of fat deposition. The latter, however, should not affect the final product as lipid extraction during further downstream processing is expected to minimize these differences.

The surface morphology of a collagen membrane is likely to affect cellular behavior such as attachment, migration, proliferation, stem cell differentiation and gene expression [29,45–47]. Therefore, an in-depth ultrastructural investigation of the membrane surfaces was performed. Electron microscopy provides a resolution in the nanometer range [48], making this technique the method of choice for studying collagen fibril structure. Furthermore, scanning electron microscopy (SEM) offers a three-dimensional view of the sample surface and thus, was adopted to image the collected tissue samples. NaOH cell digestion is a widely accepted method to remove cellular components as well as interfibrillar elements while preserving fibrils [17,39,40,49]. Our results corroborated this assertion, as collagen-specific D-periodicity was observed confirming the presence of collagen fibers.

In membranes lined with a mesothelium, the collagenous structure seen after NaOH treatment most likely represents the submesothelial connective tissue. This finding explains the ultrastructural uniformity of the surfaces of CT, PE and PL. The epithelial lining of serous membranes is known to feature mesothelial openings, which are the so-called lymphatic stomata [50]. The lymphatic stomata establish connections from the visceral cavities to the submesothelial lymphatic system [51], and the stomata have been reported on both the muscular portion and the tendinous portion of the diaphragm in different species [52,53]. Peritoneal lymphatic stomata occur in circumscribed areas where they are arranged in clusters or strips [50,52,53]. Maculae cribriformes are round or oval foramina in the submesothelial connective tissue. They are located between the peritoneal mesothelial cells and the subperitoneal lymphatic lacunae [50,54] and are considered to function as a prelymphatic pathway [54]. The discontinuities observed in circumscribed areas of the caudal face of the CT membrane are thus considered to represent maculae cribriformes, which is a structure that is essential for the absorption of molecules and cellular elements into the lymphatic system.

Compared to the membranes with a mesothelial covering, both sides of the SIS membrane were fuzzier, with one face being smoother than the opposite one. This finding is in accordance with previous reports [55], which showed that the mucosal side of SIS was smoother than the serosal (muscular) side. Rat SIS has been reported to consist of stacked collagen layers with alternating fiber orientations of ±30° relative to the longitudinal axis of the intestine [56]. Additionally, Sacks and Gloeckner [44] occasionally found these two fiber populations in porcine SIS, but the researchers postulated an overall fiber alignment parallel to the longitudinal axis of the intestine. Our own results corroborate the contention of a layered arrangement, but a preferential fiber orientation was not systematically present. However, mechanical strain during SIS processing may have distorted the original fiber orientation.

Fibril diameters were determined in SEM micrographs, which revealed statistically significant differences between some membrane types. The median fibril diameter was lowest in SIS and highest in CT, with values ranging only from 47 to 70 nm, respectively. The mean fibril diameter and fibril diameter distribution in healthy tissues are known to depend on developmental stage, age and type of tissue [22,23]. The average diameter of collagen fibrils is positively correlated with the tensile strength of a connective tissue; moreover, the fibril diameter distribution is closely related to the duration and magnitude of mechanical strain the tissue is exposed to [23]. Tissues such as flexor tendons experience a sustained mechanical load. These tissues show a bimodal or multimodal distribution of fibril diameters, ranging from several nanometers to more than 500 nanometers, which is associated with a higher tensile strength [14,18,23]. In contrast, tissues that are less stressed, such as the cornea, blood vessels or interstitial stroma, show a unimodal fibril diameter distribution. The exact diameter of these uniform fibrils varies depending on the tissue, where the range is narrow, but typically these fibrils are not larger than 100 nm [14,18]. The membranes investigated in the present study all revealed a unimodal distribution of small diameter fibrils. This finding is consistent with the limited magnitude and duration of stress levels encountered by these membranes.

Collagen fibrils are assembled from filamentous subunits called microfibrils or subfibrils [18,20,21]. Helical cleavages in a right-handed direction have been reported in corneal and scleral collagen fibrils, as shown by SEM [20]. Similar helical cleavages were frequently observed in our material as well, and although the inclination angles were not determined, the angles were on the same order of magnitude of 17–18°, as has been reported to be typical for unimodal small diameter fibrils [18,21].

The median D-period was in the range of 53–55 nm for all the membranes studied. Similarly, a mean D-periodicity between 52 and 54 nm has recently been reported for porcine pericardium collagen in a TEM study [43]. This value is somewhat less than the typically reported 63 to 70 nm for various tissues using different methods such as transmission electron microscopy (TEM) of plastic embedded tissue, scanning electron microscopy, low-angle X-ray diffraction methods or atomic force microscopy [17,20,21]. Tissue preparation obviously has the potential to affect D-periodicity [17,57], and drying the samples for SEM may have contributed to this comparatively short D-period. Our measurements revealed statistically significant differences in D-periodicity between some membrane types. However, these differences were minimal in absolute terms and, hence, are not expected to be of functional relevance. In any case, the presence of an axial periodical striation confirmed the collagenous nature of the investigated tissue fibrils.

The major limitations of electron microscopy result from the fixation and dehydration procedures biological tissues have to undergo. In the present study, the processing was exacerbated by subjecting the tissue to a digestion with NaOH. Therefore, electron microscopy was complemented by second harmonic generation (SHG) and two-photon excited fluorescence (TPEF), techniques that allow the differentiation of collagen (SHG) and elastic (TPEF) fibers in native membranes. SHG and TPEF also provide a comprehensive view of the fiber arrangement throughout a membrane. This information is a prerequisite to assessing collagen architecture, which in turn, immediately affects the mechanical behavior of the membrane [44,58,59]. The main SHG signal is transmitted forward. Depending on the molecular orientation and overall distribution of the emitters within the focal volume, some of the information, however, is backscattered [60–62]. To collect the full SHG signal, both the forward and backward signals were detected. SHG/TPEF substantiated the idea of a layered arrangement of undulating collagen fibers. In general, the collagen fibers of all the membrane types studied were arranged in layers parallel to the surface. For CT, the layering was particularly obvious, but the collagen fibers were less undulated, as has been reported by others [58,63]. As opposed to the situation in dogs [63], however, the CT membrane did not reveal two distinct orthogonal layers. In the PE membrane, the collagen fibers were strongly undulating. This finding is consistent with the reports of a more pronounced waviness of collagen fibers in pig PE than in cattle PE [43]. The observation of a well-defined stratification in *pericardium fibrosum* also substantiates previous findings [43,59,64]. Within the layers, the degree of collagen fiber alignment was variable; therefore, stacked layers with the same or alternating collagen fiber orientation, as well as layers with randomly organized fibers, were present. The multidirectional arrangement of collagen fibers in the plane parallel to the pericardial surface is congruent with the findings reported by [59,65] but differs from those of another study [43].

The presence of elastic fibers was very conspicuous in the most superficial tissue layers. This result is fully compatible with the notion of an elastic network supporting the mesothelial lining [17,66–68]. In deeper tissue layers, elastic fibers were usually less abundant, with the smallest gradient being noticed in PE. This finding is in line with the previously reported presence of elastic fibers throughout PE [69,70]. In membranes thin enough to be completely transilluminated such as the PL membrane, the elastin signal increased again toward the opposite surface. Verhoeff-van Gieson staining of histological sections corroborated these observations (https://doi.org/10.6084/m9.figshare.7022669).

Taken together, these findings indicate that the decrease in elastin signal in deeper layers does not result from signal loss. Most elastic fibers ran parallel to the collagen fibers. This finding suggests a functional interaction of these two fibrous components that imparts the tissue with both tensile strength and elasticity.

Overall, the present study provides an extensive morphological analysis of four porcine membranes with potential for use in regenerative medicine and dentistry. We expect the results to provide a rationale for selecting appropriate applications and for developing adequate processing procedures.
